# Identification, Characterization, and Virulence Gene Expression of Marine Enterobacteria in the Upper Gulf of Thailand

**DOI:** 10.3390/microorganisms10030511

**Published:** 2022-02-26

**Authors:** Pongrawee Nimnoi, Neelawan Pongsilp

**Affiliations:** 1 Department of Microbiology, Faculty of Liberal Arts and Science, Kasetsart University, Nakhon Pathom 73140, Thailand; umco_perra@hotmail.com; 2 Department of Microbiology, Faculty of Science, Silpakorn University, Nakhon Pathom 73000, Thailand

**Keywords:** enterobacteria, the Gulf of Thailand, bacterial diversity, virulence gene expression, antibiotic resistance, β-lactamase gene, silver and gold nanoparticles

## Abstract

Enterobacteria that commonly inhabit marine environments have a great impact on human health. In this study, enterobacteria isolated from seawater in the Upper Gulf of Thailand were identified and characterized. Seawater from nine sampling sites along the Upper Gulf of Thailand contained presumptive enterobacteria that ranged from 0.22 ± 0.44 to 17.00 ± 3.97 CFU/mL. The 101 strains belonged to seven species in which *Klebsiella pneumoniae* was the majority (47.5% of strains). The highest prevalence was resistant to ampicillin (76.2%) and ticarcillin (72.3%), respectively, whereas none was resistant to imipenem. Forty-five antibiotic resistance patterns were observed and 33.7% exhibited multidrug resistance, emphasizing the concern about public health. Three β-lactamase genes, including *ampC*, *blaSHV*, and *blaTEM*, were detected at the frequencies of 47.5%, 21.8%, and 11.9%, respectively. Six virulence genes, including *csgD*, *uge*, *kfu*, *eaeA*, *magA*, and *LTI*, were detected at the frequencies of 37.6%, 31.7%, 19.8%, 16.8%, 12.9%, and 5.9%, respectively. The condition of 4% NaCl downregulated the expression of the *kfu* and *uge* genes. The 67.3% and 63.4% of strains synthesized silver nanoparticles ranging between 3.04 ± 0.64 and 20.64 ± 0.95 μg/mL and gold nanoparticles ranging between 7.77 ± 0.45 and 57.57 ± 8.00 μg/mL, respectively.

## 1. Introduction

In our previous publication, the community and diversity of total bacteria present in seawater of the Upper Gulf of Thailand was first reported [1]. In this study, we further focused on culturable enterobacteria, an important group that has a large impact on human health. Enterobacteria, a heterogeneous group of the γ-*Proteobacteria*, belong to the order *Enterobacterales* and consist of eight families including *Budviciaceae*, *Enterobacteriaceae*, *Erwiniaceae*, *Hafniaceae*, *Morganellaceae*, *Pectobacteriaceae*, *Thorselliaceae*, and *Yersiniaceae*. Examples of genera include *Citrobacter*, *Enterobacter*, *Erwinia*, *Escherichia*, *Klebsiella*, *Morganella*, *Pantoea, Proteus*, *Salmonella*, *Serratia*, *Shigella*, and *Yersinia* [2,3]. Enterobacteria can cause a variety of diseases such as nonalcoholic steatohepatitis, allergy, eczema, asthma, inflammatory bowel disease, bacteremia, and neonatal meningitis [4,5,6,7]. Members of enterobacteria are considered as indicators for bacterial contamination in food and water and food safety evaluation; thus, are of sanitary significance [8]. Enterobacteria were found to commonly inhabit marine environments. Their prevalence was reported in seawater in Italy [9], Israel [10], Ireland [11,12], Brazil [13], and Palestine [14]. The contamination by enterobacteria in seawater affects water quality, and consequently endangers marine microbiota, marine animal health, and human health.

The characteristics that have been highlighted among enterobacteria include antibiotic resistance, pathogenesis, and metabolite synthesis. Antibiotic resistance has been reported to be a common characteristic among enterobacteria. Previous reports demonstrated that enterobacteria exhibited high levels of resistance to many antibiotics [15,16]. Marine enterobacteria were also found to be multidrug-resistant [14,17,18,19]. Enterobacteria have a special ability to obtain multidrug resistance in a single step by acquiring several resistance genes from various bacterial species and integrating those genes into the same plasmids [20]. This mechanism enables pathogens to become resistant to multiple antibiotics, increasing risks of failures in treatments with last resort antibiotics [21]. Trends in resistance patterns offer important insights regarding emerging pathogens as well as inform strategies for public health, infection control, and antimicrobial approaches [22]. Antibiotic resistance and virulence of pathogens play important roles in the infection process. Antibiotic resistance and virulence genes of enterobacteria are consequently of interest. Resistance and virulence are inextricably related to the phylogenetic information of the strain [23]. Even members of the same genus, only some species and strains are pathogenic [24]. Additionally, the strains (or species) differ in their virulence [25]. Virulence genes, which may be located on chromosomal pathogenicity islands (PAIs), plasmids, and other mobile genetic elements, control the disease-causing ability of bacteria [26]. Virulence genes can be classified into three types including (1) true virulence genes which encode factors or enzymes producing factors that are involved in bacteria-host interaction and directly cause the pathological damage during infection; (2) virulence-associated genes which encode factors or enzymes producing factors that control virulence gene expression or stimulate virulence factors or are essential for the activity of true virulence factors; and (3) virulence life-style genes which encode factors or enzymes producing factors that promote host colonization or enable evasion of the host immune system or enable interaction survival or employ host-factors for the advantages of survival [27]. Previous reports demonstrated that the expression of virulence genes was affected by several factors such as (1) nutrient starvation [28,29,30]; (2) osmotic agents (NaCl, sucrose, and glucose) [29,31,32,33,34,35,36]; (3) oxygen [37]; (4) temperature [31,38]; (5) growth phase [29,39,40]; (6) kind of carbon source [41,42]; (7) pH [43]; (8) sodium glycocholate [44]; and (9) L-glutamine [45]. Besides being a major problem in public health concern, enterobacteria are also highlighted for their application potential. Enterobacteria are promising nanoparticle-synthesizing bacteria based on the advantages of rapid, nonpolluted and nontoxic reaction, cost-effectiveness, easy purification of nanoparticles in supernatant, and size of nanoparticles [46,47,48,49]. Previous studies reported the ability of enterobacteria to synthesize various nanoparticles such as gold [49,50], silver [51,52], selenium [53], lead [54], and cadmium sulfide [55].

In this study, we investigated enterobacteria isolated from seawater at nine sites along the shores of the Upper Gulf of Thailand in the following aspects: (1) the use of enterobacterial repetitive intergenic consensus-polymerase chain reaction (ERIC-PCR) fingerprinting to distinguish enterobacterial strains; (2) the use of two-primers random amplified polymorphic DNA (TP-RAPD) fingerprinting to distinguish enterobacterial species; (3) the identification of enterobacterial species by using the Vitek^®^ mass spectrometry (MS) system; (4) the dominant characteristics including resistance to antibiotics as well as synthesis of silver and gold nanoparticles; (5) the detection and sequence analysis of antibiotic resistance and virulence genes; and (6) the quantitative assessment of factors affecting the expression of virulence genes by a quantitative real-time PCR (qRT-PCR).

## 2. Materials and Methods

### 2.1. Isolation of Enterobacteria from Seawater Samples

**Figure 1:**
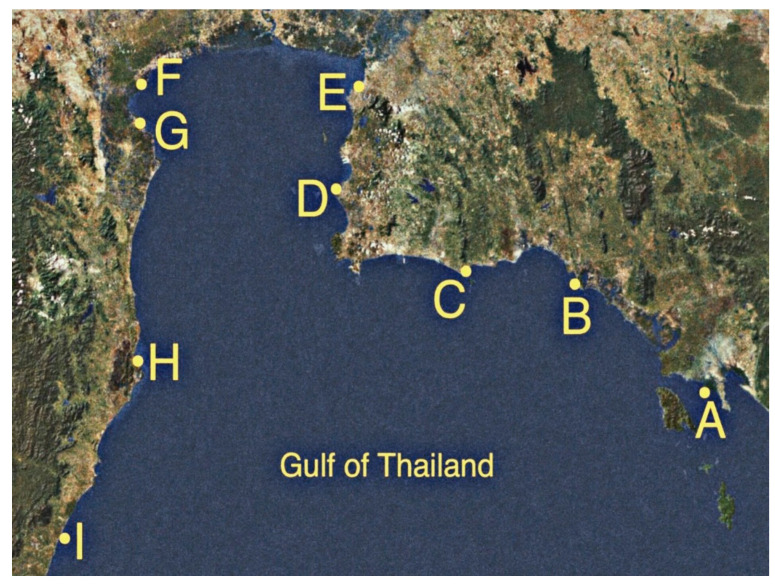
Nine sampling sites along the shores of the Upper Gulf of Thailand. Site A, Black Sand Beach; B, Kungkrabaen Bay; C, Suanson Beach; D, Pattaya Beach; E, Angsila old market; F, Donhoylhod; G, Bangtaboon Bay; H, Pranburi forest park; I, Wanakorn Beach.

Seawater was collected on 10 November and 1 December 2018 at nine sites in seven provinces along the shores of the Upper Gulf of Thailand, with a distance of approximately 769.97 km (Table 1 and Figure 1). Sampling sites were categorized into three types of land use that were presumably affected by different run-off conditions. Three mangrove forests were at Black Sand Beach (site A), Kungkrabaen Bay (site B), and Pranburi forest park (site H). Three tourist sites were at Suanson Beach (site C), Pattaya Beach (site D), and Wanakorn Beach (site I). Three aquaculture sites were at Angsila old market (site E), Donhoylhod (site F), and Bangtaboon Bay (site G). At each sampling site, near-surface seawater (depth approximately one foot) was collected approximately 2 m from the shoreline during the day (from 8 a.m. to 2 p.m.). The aseptic sampling was performed in triplicate as previously described [56] and the collected samples were stored on ice during transit. Temperatures and pH values of seawater samples from nine sampling sites ranged from 27 °C to 31 °C and 6.7 to 7.5, respectively. The isolation of enterobacteria was performed within 6 h after sample collection as previously described [57]. The numbers of presumptive enterobacteria in seawater samples were enumerated by a pour plate technique using a selective medium, violet red bile glucose (VRBG) agar (Titan Biotech. Ltd., Delhi, India) and reported as colony forming unit (CFU)/mL. Presumptive enterobacteria were selected based on the following characteristics: pink to violet-red colonies of 0.5 mm or greater in diameters on VRBG agar and gram-negative bacilli or coccobacilli that were oxidase-negative [58]. Pure cultures of presumptive enterobacterial isolates were kept both at 4 °C on Luria-Bertani (LB) slants and at −80 °C in 20% glycerol. The most-probable-number (MPN) indices of coliform bacteria were determined by the standard multiple tube fermentation method as previously described [59].

### 2.2. ERIC-PCR Fingerprinting of Presumptive Enterobacterial Isolates

ERIC-PCR was performed to assess genotypic diversity and to reveal clusters among isolates as well as to differentiate individual strains. ERIC-PCR employed genomic DNA extracted from cell pellets of each isolate as a DNA template, a pair of primers ERIC2 (5′ AAG TAA GTG ACT GGG GTG AGC G 3′) and ERIC1R (5′ ATG TAA GCT CCT GGG GAT TAC C 3′), and a thermal cycling condition consisting of a first denaturation step at 95 °C for 7 min, 30 cycles of denaturation at 95 °C for 1 min, annealing at 52 °C for 1 min, and extension at 65 °C for 8 min, and a final extension step at 65 °C for 16 min [60]. Negative controls (no DNA template) were included in PCR reactions. The amplified fragments were separated by electrophoresis on 1%% agarose gels containing SafeView FireRed gel casting dye (Applied Biological Materials Inc., Richmond, BC, Canada) and visualized with a Molecular Imager^®^ Gel DocTM XR+ system (Bio-Rad Laboratories, Hercules, CA, USA). ERIC-PCR fingerprints and clusters were presented in the unweighted pair groups using mathematical averages (UPGMA) dendrogram created with the Phoretix ID Pro. software (TotalLab Ltd., Newcastle upon Tyne, UK). The isolates generating distinct ERIC-PCR fingerprints were characterized as individual strains and then were subjected to further steps of the study.

### 2.3. TP-RAPD Fingerprinting of Presumptive Enterobacterial Strains and Species Identification by Using the Vitek^®^ MS System

TP-RAPD was performed to distinguish species among presumptive enterobacterial strains. Genomic DNA of each strain was used as a DNA template in PCR reactions using a pair of primers, 8F (5′ AGA GTT TGA TCC TGG CTC AG 3′) and 1522R (5′ AAG GAG GTG ATC CAN CCR CA 3′) (R = C/T; N = A/G/C/T). PCR cycles consisted of a first denaturation step at 95 °C for 9 min, and then 35 cycles of denaturation at 95 °C for 1 min, annealing at 45 °C for 1 min, and extension at 72 °C for 2 min, followed by a final extension step at 72 °C for 7 min [61]. Negative controls (no DNA template) were included in PCR reactions. The amplified fragments were separated and visualized as described above. A representative strain from each TP-RAPD pattern was subjected to species identification using the Vitek^®^ MS system (bioMerieux, Inc., Durham, NC, USA).

### 2.4. Examination on Antibiotic Resistance of Enterobacterial Strains

Enterobacterial strains were examined for resistance to 14 antibiotics in 6 categories as previously described [62]. The experiments were performed in triplicate and the interpretation was in accordance with a zone size interpretative chart [63]. The tested antibiotics included (1) ampicillin 10 μg; (2) ticarcillin 75 μg; (3) cefepime 30 μg; (4) cefoxitin 30 μg; (5) ceftriaxone 30 μg; (6) imipenem 10 μg; (7) meropenem 10 μg; (8) ciprofloxacin 5 μg; (9) levofloxacin 5 μg; (10) norfloxacin 10 μg; (11) amikacin 30 μg; (12) gentamicin 10 μg; (13) tobramycin 10 μg; and (14) chloramphenicol 30 μg.

### 2.5. Detection and Sequence Analysis of Antibiotic Resistance and Virulence Genes in Enterobacterial Strains

Genomic DNA of each enterobacterial strain was used as a DNA template in PCR reactions to detect the presence of ten antibiotic resistance and ten virulence genes as previously described. The antibiotic resistance genes examined were as follows: (1) *ampC* [64]; (2) *blaCMY2* [65]; (3) *blaCTX-M* [66]; (4) *blaKPC* [65]; (5) *blaNDM* [65]; (6) *blaSHV* [66]; (7) *blaTEM* [66]; (8) *blaVIM* [65]; (9) *blaZ* [67]; and (10) *mecA* [68]. The virulence genes examined were as follows: (1) *cnf2* (for synthesis of cytotoxic necrotizing factor) [69]; (2) *csgD* (for synthesis of curli) [70]; (3) *eaeA* (for interaction with host cell) [69]; (4) *espB* (for interaction with host cell) [71]; (5) *kfu* (for iron uptake system) [72]; (6) *LTI* (for synthesis of thermolabile toxin) [69]; (7) *magA* (for synthesis of capsular polysaccharide) [72]; (8) *STII* (for synthesis of thermolabile toxin) [69]; (9) *uge* (for synthesis of lipopolysaccharide) [72]; and (10) *vt2e* (for synthesis of verotoxin) [69]. Nucleotide sequences of primers and sizes of the PCR products are shown in Table S1. The presence and sizes of the amplified fragments were determined by electrophoresis on 1% agarose gels. The PCR positive bands were eluted from agarose gels using a QIAquick gel extraction kit (Qiagen, Valencia, CA, USA). The nucleotide sequences of purified PCR products were determined by Bio Basic, Inc. (Markham, ON, Canada) and then aligned with reference sequences in order to obtain percentages of sequence identity by using the BLASTn program that is available on the website of the National Center for Biotechnology Information (NCBI) (https://blast.ncbi.nlm.nih.gov/Blast.cgi) (accessed on 28 January 2022).

### 2.6. Statistical Analysis for Association between Antibiotic Resistance Genes and Virulence Genes

The data analysis was processed with SPSS statistical software version 19.0 (IBM Corp., Chicago, IL, USA). Association of antibiotic resistance genes and virulence genes were analyzed by Pearson’s and Spearman’s correlations. A *p*-value of ≤0.05 was considered indicative of significance.

### 2.7. Examination on Factors Affecting the Expression of Virulence Genes

A representative strain harboring virulence genes was subjected to examination on factors affecting gene expression by qRT-PCR analysis. Specific primers for virulence genes used in qRT-PCR experiments were designed using the Primer-BLAST program of NCBI (https://www.ncbi.nlm.nih.gov/tools/primer-blast/index.cgi?LINK_LOC=Blast Home) (Table 2). The housekeeping gene, *recA* (recombinase A), was selected because it was the most suitable reference gene for accurate reverse transcription quantitative real-time PCR data normalization in *K*. *pneumoniae* [73]. To examine the effect of NaCl concentrations on the expression of virulence genes, a representative strain was cultured in four types of medium including LB without NaCl, LB with 0.5% (wt/vol) NaCl, LB with 1% (wt/vol) NaCl, and LB with 4% (wt/vol) NaCl. Three mL of the cell cultures were centrifuged, and the cell pellets were washed with DNase-free water. Total RNA of the cultures grown under each condition was extracted by using an RNeasy mini kit (Qiagen, Valencia, CA, USA). The RNA concentration and purity were determined photometrically at 260 nm, 280 nm, and 230 nm using a Nanodrop 2000C spectrophotometer (Thermo Scientific, Waltham, MA, USA). RNA bands were examined by agarose (1% in TBE buffer) gel electrophoresis. RNA was diluted to 10 ng/μL in molecular biology grade nuclease and protease-free water (Apsalagen Co., Ltd., Bangkok, Thailand). Complementary DNA (cDNA) was synthesized from 50 ng of RNA using an IScript^TM^ reverse transcription supermix for RT-qPCR (Bio-Rad Laboratories, Hercules, CA, USA). The real-time PCR was performed with a qPCRBIO SyGreen 1-step detect Lo-Rox (PCR Biosystems Ltd., London, UK) in a CFX Connect^TM^ real-time PCR detection system (Bio-Rad Laboratories, Hercules, CA, USA). Thermal cycling included 3 min of initial denaturation at 95 °C, denaturation at 95 °C for 10 sec, annealing/extension at 60 °C for 30 sec, and fluorescence acquisition at the end of each extension for 39 cycles. Melting curve measurements were performed after the completion of cycling.

### 2.8. Examination on Nanoparticle Synthesis by Enterobacterial Strains

The synthesis of silver and gold nanoparticles by enterobacterial strains was investigated. All strains were cultured in LB at 37 °C with shaking at 120 rpm for 40 h. Cultures were centrifuged at 11,000 rpm for 10 min at 4 °C in an Eppendorf 5804R centrifuge (Eppendorf, Selangor Darul Ehsan, Malaysia). Five mL of supernatant was mixed with the solutions of 1 mM AgNO_3_ (for silver nanoparticles) and 1 mM HAuCl_4_·3H_2_O (for gold nanoparticles) and the resulting mixtures were allowed to stand at 55 °C for 48 h in the dark. The color changes from light-yellow to brown and from light-yellow to pink, red, and purple indicated the synthesis of silver and gold nanoparticles, respectively [74,75]. The visible ultraviolet (UV-vis) spectra of silver and gold nanoparticles were determined using a Nanodrop 2000C spectrophotometer (Thermo Scientific, Waltham, MA, USA). The optical densities (ODs) at λmax were measured with a Cecil CE1011 spectrophotometer (Cecil Instruments, Cambridge, UK). The concentrations of silver and gold nanoparticles were evaluated from the standard curves of silver and gold nanoparticles with the same λmax (Sigma-Aldrich, St. Louis, MO, USA), respectively.

## 3. Results

### 3.1. Prevalence of Enterobacteria in Seawater along the Upper Gulf of Thailand

Seawater parameters, including temperature, pH, salinity, turbidity, total suspended solid (TSS), total N, total P, and five-day biochemical oxygen demand (BOD_5_) at nine sampling sites along the shores of the Upper Gulf of Thailand, were reported in our previous study [1]. Seawater from all sites had the same salinity value of 4.0% NaCl, except sites F (aquaculture site at Donhoylhod) and G (aquaculture site at Bangtaboon Bay) that had the salinity values of 3.0% NaCl and 1.0% NaCl, respectively. Seawater from nine sampling sites contained presumptive enterobacteria that ranged from 0.22 ± 0.44 to 17.00 ± 3.97 CFU/mL. Numbers of total coliform bacteria ranged from 4.5 to 3500 MPN index/100 mL. Average number of presumptive enterobacteria and number of total coliform bacteria in each sampling site are shown in Table 3. The 129 presumptive enterobacterial isolates were derived and designated by abbreviations GTH followed by the letters A-I that indicate the sites according to those presented in Table 1 and then followed by the isolate number.

### 3.2. ERIC-PCR Fingerprinting of Presumptive Enterobacterial Isolates

The 113 distinct ERIC-PCR patterns were generated by the 129 presumptive enterobacterial isolates, suggesting that there were 113 individual strains. These ERIC-PCR patterns were distinguished based on the number and size of the amplified bands that ranged from one to seven and from approximately 100 to 5000 bp, respectively. The UPGMA dendrogram (Figure S1) presents genetic relatedness among strains. The isolates with 100% similarity of ERIC-PCR fingerprints were defined as the same strains. Presumptive enterobacterial strains were selected for subsequent studies.

### 3.3. Distinguishing Presumptive Enterobacterial Species Based on TP-RAPD Fingerprinting

**Figure 2:**
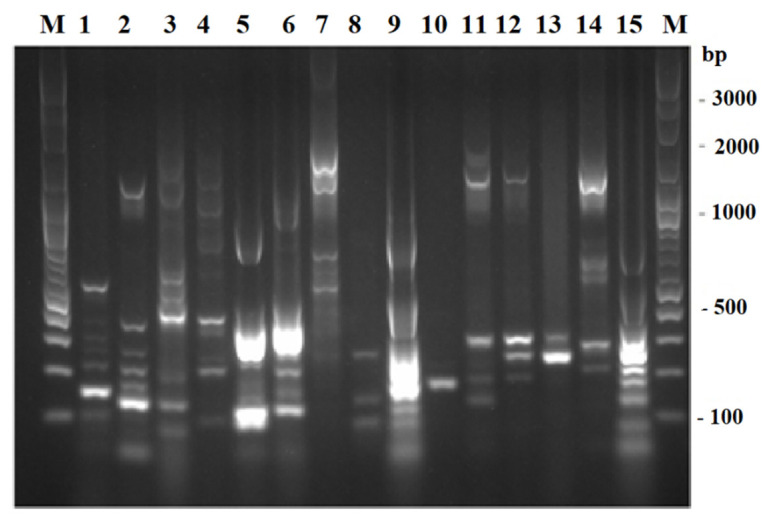
TP-RAPD fingerprints of 113 presumptive enterobacterial strains. Lane M: 100 bp plus DNA ladder; 1–15: 15 different TP-RAPD fingerprints.

As depicted in Figure 2, 15 distinct TP-RAPD patterns were generated from 113 presumptive enterobacterial strains, suggesting that these strains belonged to 15 species. The amplified bands ranged in number from one to eight and in size from approximately 100 to 2000 bp.

### 3.4. Species Identification of Presumptive Enterobacterial Strains Based on the Vitek^®^ MS System

The Vitek^®^ MS system was employed for species identification of 15 representative strains belonging to each TP-RAPD pattern. These strains were identified (with 99.2 to 99.9% confidence values) as being members of 15 species including (1) *K*. *pneumoniae* (48 strains out of 113; 42.5%); (2) *Escherichia coli* (16 strains; 14.2%); (3) *Enterobacter cloacae* (13 strains; 11.5%); (4) *Klebsiella variicola* (12 strains; 10.6%); (5) *Enterobacter hormaechei* (seven strains; 6.2%); (6) *Klebsiella aerogenes* (four strains; 3.5%); (7) *Aeromonas caviae* (two strains; 1.8%); (8) *Sphingomonas paucimobilis* (two strains; 1.8%); (9) *Vibrio fluvialis* (two strains; 1.8%); (10) *Vibrio vulnificus* (two strains; 1.8%); (11) *Pantoea* sp. (one strain; 0.9%); (12) *Photobacterium damselae* (one strain; 0.9%); (13) *Pseudomonas mendocina* (one strain; 0.9%); (14) *Vibrio alginolyticus* (one strain; 0.9%); and (15) *Vibrio parahaemolyticus* (one strain; 0.9%). Among 113 presumptive enterobacterial strains, 101 strains (89.4%) were enterobacteria in the genera *Enterobacter*, *Escherichia*, *Klebsiella*, and *Pantoea*.

### 3.5. Prevalence of Antibiotic Resistance among Enterobacterial Strains 

Forty-five antibiotic resistance patterns, as shown in Table S2, were observed among 101 enterobacterial strains. The most common resistance pattern (14.9%) was the ampicillin and ticarcillin co-resistance. Six strains (5.9%) were susceptible to all 14 antibiotics tested. The remaining 95 strains were resistant to at least one antibiotic except imipenem. The resistance to ampicillin, ticarcillin, ciprofloxacin, norfloxacin, cefoxitin, tobramycin, chloramphenicol, cefepime, amikacin, gentamicin, levofloxacin, ceftriaxone, and meropenem was found in 77 (76.2%), 73 (72.3%), 45 (44.6%), 41 (40.6%), 32 (31.7%), 15 (14.9%), 15 (14.9%), 12 (11.9%), 7 (6.9%), 6 (5.9%), 5 (5.0%), 4 (4.0%), and 3 (3.0%) strains, respectively, while none was resistant to imipenem. *E*. *coli* GTH-F3 was resistant to most (11) antibiotics including ampicillin, ticarcillin, cefepime, cefoxitin, ceftriaxone, ciprofloxacin, levofloxacin, norfloxacin, amikacin, gentamicin, and tobramycin. Among 64 *Klebsiella* strains, the resistance to ticarcillin (85.9% of strains), ampicillin (82.8%), ciprofloxacin (53.1%), norfloxacin (50.0%), cefoxitin (18.8%), chloramphenicol (17.2%), cefepime (14.1%), tobramycin (12.5%), amikacin (7.8%), gentamicin (6.3%), ceftriaxone (4.7%), meropenem (4.7%), and levofloxacin (4.7%) was observed. Among 20 *Enterobacter* strains, the highest percentage of resistant strains was found with cefoxitin (80.0%), followed by ampicillin (75.0%), ticarcillin (40.0%), meropenem (20.0%), norfloxacin (20.0%), cefepime (5.0%), amikacin (5.0%), gentamicin (5.0%), and tobramycin (5.0%). Among 16 *E*. *coli* strains, the percentages of resistant strains were as follows: ticarcillin (56.3%), ampicillin (50.0%), ciprofloxacin (37.5%), tobramycin (37.5%), norfloxacin (31.3%), cefoxitin (25.0%), levofloxacin (12.5%), ceftriaxone (6.3%), cefepime (6.3%), amikacin (6.3%), gentamicin (6.3%), and chloramphenicol (6.3%). *Pantoea* sp. was resistant to ampicillin, ticarcillin, cefepime, and ciprofloxacin.

The 14 antibiotics tested were sorted into six categories including (1) penicillin (ampicillin and ticarcillin); (2) cephalosporin (cefepime, cefoxitin, and ceftriaxone); (3) carbapenem (imipenem and meropenem); (4) monobactam (ciprofloxacin, levofloxacin, and norfloxacin); (5) aminoglycoside (amikacin, gentamicin, and tobramycin); and (6) phenicol (chloramphenicol). Multidrug resistant (MDR) is defined as nonsusceptibility to at least one agent in three or more antimicrobial categories after removing the agent or category which a species has intrinsic resistance [76]. Therefore, 34 strains (33.7%), including *K*. *pneumoniae* (16 strains; 15.8%), *K*. *variicola* (six strains; 5.9%), *E*. *coli* (five strains; 5.0%), *E*. *hormaechei* (four strains; 4.0%), *K*. *aerogenes* (two strain; 2.0%), and *Pantoea* sp. (one strain; 1.0%), were classified as MDR.

### 3.6. Detected Antibiotic Resistance Genes in Enterobacterial Strains

Positive bands with sizes compatible with the presence of three antibiotic resistance genes, including *ampC*, *blaSHV*, and *blaTEM*, were detected by PCR reactions. Sequencing of PCR-amplified fragments from representative strains reinforced the presence of these genes based on 95.61 to 100.00% identities to the reference sequences. The sequences of antibiotic resistance genes were deposited in the NCBI database under GenBank accession numbers OM460077-OM460087. Seven antibiotic resistance gene patterns were observed among 59 enterobacterial strains (58.4% of strains) while the remaining 42 strains provided negative results for the presence of all ten antibiotic resistance genes. The number of positive strains in each genus or species that possessed antibiotic gene is shown in Table 4. Antibiotic resistance gene patterns included (1) *ampC* (28 strains; 27.7%); (2) *blaSHV* (six strains; 5.9%); (3) *blaTEM* (three strains; 3.0%); (4) *ampC blaSHV* (13 strains; 12.9%); (5) *ampC blaTEM* (six strains; 5.9%); (6) *blaSHV blaTEM* (two strains; 2.0%); and (7) *ampC blaSHV blaTEM* (one strain; 1.0%). The frequency of the *ampC* gene was highest (48 strains; 47.5%), followed by the *blaSHV* gene (22 strains; 21.8%), and the *blaTEM* gene (12 strains; 11.9%), respectively. These genes encode β-lactamases that confer resistance to the β-lactam group of antibiotics such as ampicillin and ticarcillin. The co-occurrence of three β-lactamase genes, including *ampC*, *blaSHV*, and *blaTEM* in a single strain, was noticed in *K*. *pneumoniae* GTH-A4.

### 3.7. Detected Virulence Genes in Enterobacterial Strains

Positive bands with sizes compatible with the presence of six virulence genes, including *csgD*, *eaeA*, *kfu*, *LTI*, *magA*, and *uge*, were detected by PCR reactions. Sequencing of PCR-amplified fragments from representative strains reinforced the presence of these genes based on 95.83 to 100.00% identities to the reference sequences. The sequences of virulence genes were deposited in the NCBI database under GenBank accession numbers OM460088-OM460096. A total of 17 virulence gene patterns were observed among 74 enterobacterial strains (73.3%) while the remaining 27 strains provided negative results for the presence of all ten virulence genes. The number of positive strains in each genus or species that possessed virulence gene is shown in Table 5. The 17 virulence gene patterns included (1) *csgD* (22 strains; 21.8%); (2) *eaeA* (three strains; 3.0%); (3) *LTI* (two strains; 2.0%); (4) *uge* (seven strains; 6.9%); (5) *csgD eaeA* (11 strains; 10.9%); (6) *csgD uge* (five strains; 5.0%); (7) *eaeA uge* (one strain; 1.0%); (8) *kfu LTI* (two strains; 2.0%); (9) *kfu* *magA* (one strain; 1.0%); (10) *kfu uge* (four strains; 4.0%); (11) *LTI uge* (two strains; 2.0%); (12) *magA uge* (one strain; 1.0%); (13) *csgD kfu* *magA* (one strain; 1.0%); (14) *csgD kfu uge* (one strain; 1.0%); (15) *eaeA kfu uge* (one strain; 1.0%); (16) *kfu magA uge* (nine strains; 8.9%); and (17) *eaeA kfu magA uge* (one strain; 1.0%). The frequency of the *csgD* gene was highest (37.6%), followed by the *uge* gene (31.7%), the *kfu* gene (19.8%), the *eaeA* gene (16.8%), the *magA* gene (12.9%), and the *LTI* gene (5.9%), respectively.

### 3.8. Association of Antibiotic Resistance Genes and Virulence Genes

Association of antibiotic resistance genes (*ampC*, *blaSHV*, and *blaTEM*) and virulence genes (*csgD*, *eaeA*, *kfu*, *LTI*, *magA*, and *uge*) were analyzed. The statistical analysis revealed positive and negative correlations between the two groups of genes (Table 6). The *ampC* gene exhibited significantly positive correlations with the *uge* and *magA* genes, whereas it exhibited a significantly negative correlation with the *csgD* gene. The *blaSHV* gene had the most significant positive association with the *uge* gene but negative association with the *csgD* gene. However, the *blaTEM* gene had the most significant positive association with the *eaeA* gene only. The *LTI* gene was the only one that exhibited a positive correlation with all antibiotic resistance genes.

**Figure 3:**
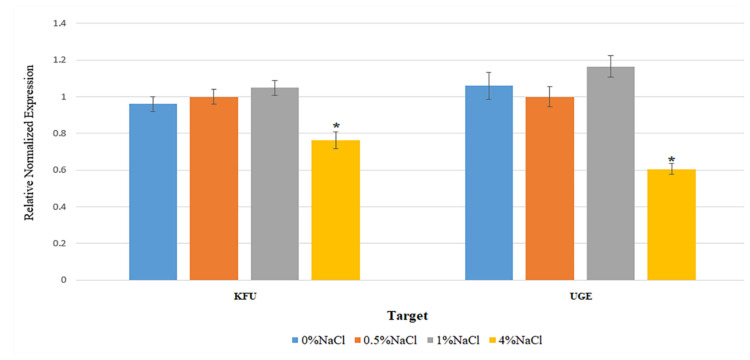
Relative gene expression levels of the virulence genes, *kfu* and *uge*, of *K*. *pneumoniae* GTH-A21 under different NaCl concentrations. Graph bars represent the means of three experiments. Error bars indicate standard deviations. * Error bars indicate standard deviations.

The strain *K*. *pneumoniae* GTH-A21 was selected to quantify the expression of two virulence genes, *kfu* and *uge*, under different NaCl concentrations (0%, 0.5%, 1%, and 4% wt/vol) by qRT-PCR. The reference gene, *recA*, was selected as being the most suitable reference gene for accurate qRT-PCR data normalization in *K*. *pneumoniae* [73]. Transcription levels determined under each condition were normalized to the internal reference gene *recA*. The relative gene expression levels (merged from three experiments) of both virulence genes, *kfu* and *uge*, of *K*. *pneumoniae* GTH-A21 under different NaCl concentrations are shown in Figure 3. The condition of 4% NaCl suppressed the expression of both *kfu* and *uge* genes. Gene expression levels under the conditions of 0% and 0.5% NaCl were not significantly different for both *kfu* and *uge* genes. Gene expression levels under the conditions of 0% and 1% NaCl were significantly different for only the *kfu* gene, whereas gene expression levels under the conditions of 0.5% and 1% NaCl were significantly different for only the *uge* gene. This study demonstrates the impact of NaCl concentration, an important physicochemical characteristic of seawater, on the expression of virulence genes in the marine bacterium, *K*. *pneumoniae*. The condition of 4% NaCl was detected in all three mangrove forests, all three tourist sites, and one aquaculture site, suggesting that marine habitats may provide the possibility to reduce pathogenicity or virulence caused by enterobacteria.

### 3.9. Synthesis of Silver and Gold Nanoparticles by Enterobacterial Strains

The 101 enterobacterial strains were examined for their ability to synthesize silver and gold nanoparticles. When the supernatant was incubated with 1 mM AgNO_3_ solution, the synthesis of silver nanoparticles was monitored visually by the change in color of the reaction solution from light-yellow to brown [74]. When the supernatant was incubated with 1 mM AuHCl_4_·3H_2_O solution, the synthesis of gold nanoparticles was monitored visually by the change in color of the reaction solution from light-yellow to red to purple-blue [75]. The λmax of silver and gold nanoparticles were 420 and 550 nm, respectively. Concentrations of synthesized silver and gold nanoparticles were calculated from standard curves constructed by using standard silver nanoparticles with λmax of 420 nm and standard gold nanoparticles with λmax of 550 nm (Sigma-Aldrich, St. Louis, MO, USA). Sixty-eight silver nanoparticle-synthesizing strains (67.3%) belonged to six species including *K*. *pneumoniae* (31 strains), *E*. *coli* (11 strains), *E*. *cloacae* (nine strains), *K*. *variicola* (eight strains), *E*. *hormaechei* (six strains), and *K*. *aerogenes* (three strains). These strains synthesized silver nanoparticles in a range of concentrations 3.04 ± 0.64 to 20.64 ± 0.95 μg/mL. Sixty-four gold nanoparticle-synthesizing strains (63.4%) belonged to seven species including *K*. *pneumoniae* (25 strains), *E*. *cloacae* (12 strains), *E*. *coli* (11 strains), *K*. *variicola* (eight strains), *E*. *hormaechei* (four strains), *K*. *aerogenes* (three strains), and *Pantoea* sp. (one strain). These strains synthesized gold nanoparticles in a range of concentrations 7.77 ± 0.45 to 57.57 ± 8.00 μg/mL. *E*. *coli* GTH-E1 and *K*. *variicola* GTH-A23 synthesized the highest amounts of silver and gold nanoparticles, respectively.

## 4. Discussion

Seawater has been found to be a reservoir of enterobacteria. For examples, *Enterobacteriaceae* were always detected in North Tyrrhenian Sea, Italy, during a 2-year period. Moreover, in 13 months, they were the major taxons with relative abundance in the range 2.5–18.1% [9]. New Delhi metallo-β-lactamase (NDM)-producing *K*. *pneumoniae* and carbapenemase-producing *Enterobacteriaceae* (CPE) were detected in seawater samples in Ireland [11,12]. *Enterobacteriaceae* accounted for 48% of 285 isolates of gram-negative bacilli from seawater in Brazil [13] and 46% of 377 isolates from seawater in Palestine [14]. The average numbers of enterobacteria and total coliforms in seawater from nine sites along the Upper Gulf of Thailand was 5.71 CFU/mL and 924.87 MPN/100 mL, respectively. In comparison, the average number of total coliforms in seawater from eight stations along the Persian Gulf was 1238.13 MPN/100 mL [77].

The presumptive enterobacterial strains were distinguished by ERIC-PCR which has been found to be extremely sensitive and can detect minor differences among strains of the same bacterial genus and species [29]. The UPGMA dendrogram (Figure S1) constructed from ERIC-PCR fingerprints presents a high level of diversity among 113 strains as showing 20% similarity. As further determined by TP-RAPD, 15 different species were distinguished. TP-RAPD was developed for taxonomy purpose as the patterns of strains in the same species are identical [78]. These 15 species were then identified and seven enterobacterial species were revealed based on the Vitek^®^ MS system which was reported as a simple, convenient, and accurate method for routine bacterial identification [79]. The Vitek^®^ MS system also provided correct identification for 99.1% of 2246 bacterial isolates [80] and 84.2% of 95 bacterial isolates [81].

In this study, 101 enterobacterial strains exhibited 45 antibiotic resistance patterns, indicating a high diversity of resistance. Only 5.9% of the strains were susceptible to all antibiotic tested while 94.1% were resistant to at least one but up to 11 of 14 antibiotics. The ampicillin and ticarcillin co-resistance was the pattern mostly found (14.9% of strains) and 33.7% were MDR. The prevalence of MDR enterobacteria in marine environment was previously reported. Approximately 35% of 1351 enterobacterial isolates from seawater samples in the eastern Adriatic Sea in Croatia were MDR [82]. Enterobacteria have a special ability to obtain multidrug resistance in a single step by acquiring several resistance genes from various bacterial species and integrating those genes into the same plasmids [20]. Multidrug resistance of enterobacteria is a challenge facing the global public health agenda.

The majority of strains (83.2%) were resistant to either or both ampicillin and ticarcillin, all of which are categorized in the penicillin group. As the major mechanism of resistance against β-lactam antibiotics (such as ampicillin and ticarcillin) in gram-negative bacteria is the synthesis of β-lactamases, which irreversibly cleave the β-lactam ring of antibiotics [83], the presence of ten β-lactamase genes was subsequently studied. The three β-lactamase genes, including *ampC*, *blaSHV*, and *blaTEM*, were detected. The 59 enterobacterial strains (58.4%) exhibited seven antibiotic resistance gene patterns while the remaining 42 strains yielded negative results for the presence of all ten β-lactamase genes. These three antibiotic genes were the same as those detected in 81 enterobacterial strains isolated from seafood in Thailand in which the *blaTEM*, *ampC*, and *blaSHV* genes were detected at the frequencies of 43%, 27%, and 24%, respectively [84].

The six virulence genes, including *csgD*, *eaeA*, *kfu*, *LTI*, *magA*, and *uge*, were detected and 17 virulence gene patterns were observed among 74 enterobacterial strains (73.3% of strains) while the remaining 27 strains provided negative results for the presence of all ten virulence genes. *K*. *pneumoniae* GTH-A6 harbored the most virulence genes including *eaeA*, *kfu*, *magA*, and *uge*. The possession of virulence genes offers a suitable approach to risk evaluation of the pathogenic potential of bacteria. Previous studies described the roles of six virulence genes detected in this study in disease progression. The *csgD* gene that encodes the synthesis of curli was reported to have a great impact on the natural phenotype of *Salmonella* such as the rdar morphotype that is beneficial to the passage through the intestinal epithelial cells [85]. The iron uptake system coding gene *kfu,* and lipopolysaccharide-synthesizing gene *uge,* were reported to be important for the invasion of mastitis-causing *K*. *pneumoniae* strains [86]. The thermolabile toxin-coding gene, *LTI* was reported as a particular gene that controlled the virulence of diarrhea-causing enterotoxigenic *E*. *coli* (ETEC) [87]. The *eaeA* gene encoding intimin that adheres to intestinal mucosa and causes intestinal lesion formation [88] was found to be associated with the severity of human disease, especially hemolytic uremic syndrome (HUS) [89]. Possession of the *eaeA* gene was common in Shiga toxin-producing *E*. *coli* (STEC) serotypes that are commonly implicated in severe human disease and outbreaks including STEC O157:H7, STEC O145:H28, and STEC O103:H2 [90]. The *magA* gene that encodes the synthesis of capsular polysaccharide is an essential virulence gene for *K*. *pneumoniae* strains. It was detected in a vast majority of *K*. *pneumoniae* liver abscess isolates and associated with resistance to killing by human serum and phagocytosis [91]. A previous study [92] demonstrated the association between the *magA* gene and endophthalmitis.

The antibiotic resistance genes and virulence genes displayed either positive or negative correlations. The *uge* gene was significantly more prevalent with the *ampC* and *blaSHV* genes. On the contrary, the *csgD* gene exhibited a significantly higher negative correlation with the *ampC* and *blaSHV* genes. The *eaeA* was the only virulence gene that was found to be positively significant with the *blaTEM* gene. Significant association of virulence genes with either antibiotic/antimicrobial resistance or antibiotic resistance gene was previously reported. The *hlyA* was the only virulence gene that was significantly more prevalent in the highly antibiotic resistant *E. coli* isolates. Three virulence genes, including *ompA*, *malX,* and *hlyA*, were obviously more abundant in the antibiotic resistant *E. coli* isolates in comparison with susceptible isolates. The *papC* gene was associated with amoxicillin resistance of *E. coli* [93]. Significant association was observed between the *iucC* gene and resistance to ampicillin and amoxicillin and between the *fimH* gene and resistance to aztreonam in Pakistani uropathogenic *E. coli* isolates [94]. Significant strong positive association between the *colM* gene for colicin resistance and the virulence genes, including *colE1*, *colB*, *traT*, and *csgA*, was observed among pathogenic *E. coli* isolated from Egyptian patients [95]. Significant association between antibiotic resistance and virulence genes was found only in *Salmonella* isolates which were resistant to more than five antibiotics and the associated virulence genes included *sefA*, *sodC1*, *sopE1*, *spvC*, *pefA*, and one integron-associated integrase class 1 gene [96].

This study examined the effect of NaCl concentrations on the expression of virulence genes (*kfu* and *uge*) by using qRT-PCR. The *kfu* gene has a role in iron acquisition. It is essential for transporting Fe^3^^+^ into cytoplasm [97]. Its contribution to capsule formation, hypermucoviscosity, purulent tissue infection, and intestinal colonization was suggested [98]. The *uge* gene has a role in capsule synthesis, resistance of phagocytosis, liver abscess, and blood infection [99]. For both *kfu* and *uge* genes, the condition of 4% NaCl suppressed the expression and the conditions of 0% and 0.5% NaCl did not differently affect the expression. The qRT-PCR has become one of the most used techniques for gene expression analysis because of its advantages of high specificity and sensitivity [100]. Even though quantification of gene expression depends on several factors such as quality of RNA, efficiency of primers, and synthesis and quantity of amplified cDNA template, the expression level of the target gene can be normalized against stably expressed internal genes to compensate for variations [101]. The bacterial pathogen senses and adapts to the prevailing conditions by modulating its gene expression [102]. The effects of NaCl concentrations on the expression of the other virulence genes were previously reported. The NaCl concentrations (<0.15% and 3.6% wt/wt) affected the expression of virulence-related genes in *Listeria monocytogenes*. The low NaCl concentration decreased the expression of the *agrA*, *ami*, *gadC*, and *opuC* genes, which are associated with osmotic stress responses, in both strains that exhibited different tolerances to salt stress [33]. The NaCl concentrations in LB medium differently affected the expression of virulence genes in a strain of *Salmonella enterica*. The condition of 1 M NaCl triggered significant overexpression of the *sopA* gene encoding E3 ubiquitin ligase, compared with that of 0 M, 0.3 M, and 0.6 M NaCl, whereas the higher NaCl concentrations (0.3 M, 0.6 M, and 1 M) significantly downregulated the expression of the *hilA* gene, a promotor of the *Salmonella* pathogenicity island [103].

The λmax of silver and gold nanoparticles synthesized by enterobacterial strains were 420 and 550 nm, respectively. The λmax of silver and gold nanoparticles were reported to be in a range of 307 to 448 nm and 400 to 700 nm, respectively, depending on size and shape [51]. The UV-vis spectroscopy is a very useful and reliable technique for the primary characterization of synthesized nanoparticles. This technique is fast, easy, simple, sensitive, selective for different types of nanoparticles, and needs only a short period for measurement [104]. The synthesis of silver and gold nanoparticles was observed with 67.3% and 63.4% of strains. Among silver and gold nanoparticle-synthesizing bacteria, enterobacteria offer several advantages including rapid, nonpolluted and nontoxic reaction, cost-effectiveness, easy purification of nanoparticles in supernatant, and size of nanoparticles. The antibacterial, antifungal, antiviral, anti-inflammatory, anti-cancer, and anti-angiogenic properties of silver nanoparticles were verified. Silver nanoparticles have been used extensively in various application perspectives such as biomedical, food, industrial, and environmental sciences [104]. Gold nanoparticles exhibit valuable properties such as surface plasmon resonance (SPR), wide surface chemistry, high binding affinity, good biocompatibility, enhanced solubility, and tunable functionalities for targeted delivery. Therefore, they can be applied to a wide range of applications including medicine, material science, biology, chemistry, and physics [105]. The enterobacterial strains examined in this study might be promising for such application in the nanotechnology.

In the present study, enterobacterial isolates of tropical marine environment were characterized, identified, and screened for the virulence-related characteristics: antibiotic resistance, presence of antibiotic resistance and virulence genes, and expression of virulence genes. The majority were resistant to ampicillin (76.2%) and ticarcillin (72.3%) which are categorized in the penicillin group. Notably, carbapenem-resistant Enterobacteriaceae (CRE) which are a major cause of concern in public health were also detected at the lowest prevalence as 3% of strains were resistant to meropenem. High proportion of strains (33.7%) were MDR and one strain (*K. aerogenes* GTH-G17) was resistant to at least one antibiotic in all six categories including penicillin, cephalosporin, carbapenem, monobactam, aminoglycoside, and phenicol. A total of 58.4% and 73.3% of strains harbored at least one antibiotic resistance and virulence gene, respectively. All three detected antibiotic resistance genes (*ampC*, *blaSHV*, and *blaTEM*) are plasmid-mediated β-lactamase genes that emerged in gram-negative bacteria. The *blaSHV* and *blaTEM* genes that encode extended-spectrum β-lactamases (ESBLs) confer resistance to a variety of antibiotics including those in penicillin, cephalosporin, and monobactam groups, causing difficulties in treatment and infection control [106]. The occurrence of CRE and MDR strains as well as possession of ESBL and virulence genes determine public health risks and might draw more attention to public health surveillance.

## Figures and Tables

**Table 1:** Site locations, sampling dates and land use.

Site	Sampling Date	Place	District, Province	Latitude	Longitude	Distance (km)	Land Use
A	11 October 2018	Black Sand Beach	Laemngop, Trat	12.169° N	102.406° E	0.00	Mangrove forest
B	11 October 2018	Kungkrabaen Bay	Thamai, Chanthaburi	12.573° N	101.902° E	123.57	Mangrove forest
C	11 October 2018	Suanson Beach	Mueang, Rayong	12.458° N	101.473° E	190.51	Tourist site
D	11 October 2018	Pattaya Beach	Banglamoong, Chonburi	12.936° N	100.883° E	326.22	Tourist site
E	11 October 2018	Angsila old market	Mueang, Chonburi	13.341° N	100.926° E	388.60	Aquaculture site
F	12 January 2018	Donhoylhod	Mueang, Samutsongkhram	13.361° N	100.022° E	527.64	Aquaculture site
G	12 January 2018	Bangtaboon Bay	Banlaem, Phetchaburi	13.264° N	99.945° E	548.77	Aquaculture site
H	12 January 2018	Pranburi forest park	Pranburi, Prachuapkhirikhan	12.412° N	99.981° E	659.23	Mangrove forest
I	12 January 2018	Wanakorn Beach	Thubsakae, Prachuapkhirikhan	11.635° N	99.703° E	769.97	Tourist site

**Table 2:** Nucleotide sequences of primers for real-time PCR and sizes of the PCR products obtained from each pair of primers.

Gene	Nucleotide Sequence of Primer	Size of the PCR Product (bp)
*kfu*	KFU-RTf 5′ CGA CCG GTT TCT GGG CGT TA 3′ KFU-RTr 5′ GGC GTT TCA AAA CCG GCG AG 3′	293
*uge*	UGE-RTf 5′ CTC TCA ACG GTC CAG TCG GC 3′ UGE-RTr 5′ CCT GTA TGC CGC CAC CAA GA 3′	288
*recA*	RECA-RTf 5′ TTA AAC AGG CCG AAT TCC AG 3′ RECA-RTr 5′ CCG CTT TCT CAA TCA GCT TC 3′	99

**Table 3:** Average numbers of presumptive enterobacteria and numbers of total coliform bacteria in sampling sites.

Site	Average Number of Presumptive Enterobacteria (CFU/mL) *	Number of Total Coliform Bacteria (MPN Index/100 mL)
A, Black Sand Beach	6.22 ± 2.68	3500
B, Kungkrabaen Bay	0.44 ± 0.73	9.3
C, Suanson Beach	17.00 ± 3.97	540
D, Pattaya Beach	1.11 ± 1.05	1600
E, Angsila old market	6.22 ± 4.58	350
F, Donhoylhod	12.33 ± 6.80	700
G, Bangtaboon Bay	6.00 ± 1.41	920
H, Pranburi forest park	1.89 ± 1.36	700
I, Wanakorn Beach	0.22 ± 0.44	4.5

* Values are means from three replicates ± standard deviations.

**Table 4:** Number of enterobacterial strain in each genus or species that possessed antibiotic gene.

Genus/Species	Number of Strain	Number of Strain Possessing Antibiotic Gene		
		*ampC*	*blaSHV*	*blaTEM*
*E* *. cloacae*	13	4	1	1
*E* *. hormaechei*	7	1	1	0
*E* *. coli*	16	4	3	6
*K* *. aerogenes*	4	1	0	0
*K* *. pneumoniae*	48	31	12	5
*K* *. variicola*	12	7	4	0
*Pantoea* sp.	1	0	1	0
Total	101	48	22	12

**Table 5:** Number of enterobacterial strain in each genus or species that possessed virulence gene.

Genus/Species	Number of Strain	Number of Strain Possessing Virulence Gene					
		*csgD*	*eaeA*	*kfu*	*LTI*	*magA*	*uge*
*E* *. cloacae*	13	8	2	0	0	0	0
*E* *. hormaechei*	7	4	0	0	0	0	1
*E* *. coli*	16	9	7	0	1	0	0
*K* *. aerogenes*	4	1	0	0	0	0	0
*K* *. pneumoniae*	48	12	7	15	2	11	23
*K* *. variicola*	12	4	1	5	2	2	7
*Pantoea* sp.	1	0	0	0	1	0	1
Total	101	38	17	20	6	13	32

**Table 6:** Nonparametric correlation analysis of antibiotic resistance genes and virulence genes.

Virulence Gene	Antibiotic Resistance Gene					
	*ampC*		*blaSHV*		*blaTEM*	
	*r_(s)_*	*p*	*r_(s)_*	*p*	*r_(s)_*	*p*
*csgD*	−0.203	0.042 *	−0.231	0.020 *	0.141	0.161
*eaeA*	−0.004	0.967	−0.109	0.277	0.244	0.014 *
*kfu*	0.179	0.074	0.068	0.502	−0.171	0.087
*LTI*	0.012	0.920	0.07	0.485	0.037	0.712
*magA*	0.285	0.004 *	−0.06	0.554	−0.141	0.159
*uge*	0.332	0.001 *	0.259	0.009 *	−0.119	0.238

*r_(s)_* Spearman’s correlation coefficient; *p*, *p*-value; * correlation is significant at the 0.05 level.

## Data Availability

All data generated or analyzed during this study have been included in this published article. Sequence data have been deposited in the NCBI database under accession numbers OM460077-OM460096.

## Supplementary Materials

The following supporting information can be downloaded at: https://www.mdpi.com/article/10.3390/microorganisms10030511/s1, Figure S1: Dendrogram constructed from ERIC-PCR patterns of the 129 presumptive enterobacterial isolates.; Table S1: Nucleotide sequences of primers and sizes of the PCR products for the examined antibiotic resistance and virulence genes.; Table S2: Antibiotic resistance patterns of enterobacterial strains and numbers of strain(s) belonging to each pattern.
