# Supplementary material for: Identification, Characterization, and Virulence Gene Expression of Marine Enterobacteria in the Upper Gulf of Thailand
Source: Microorganisms. 2022 Feb 26;10(3):511. doi: 10.3390/microorganisms10030511 (PMC8952428; doi:10.3390/microorganisms10030511)
Supplement: Supplementary file 1 [file microorganisms-10-00511-s001.zip › Figure S1.pdf]

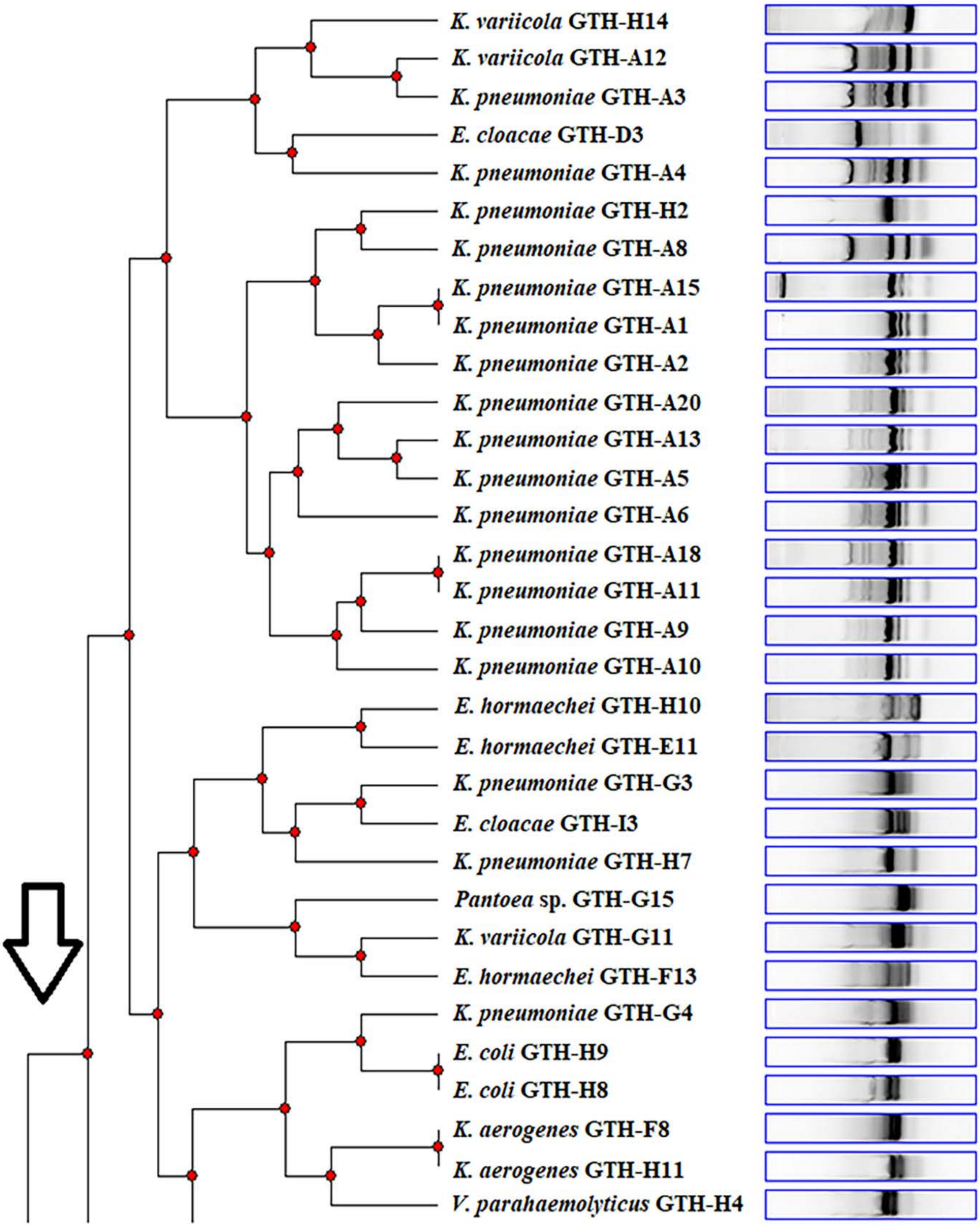
K. variicola GTH-H14
K. variicola GTH-A12
K. pneumoniae GTH-A3
E. cloacae GTH-D3
K. pneumoniae GTH-A4
K. pneumoniae GTH-H2
K. pneumoniae GTH-A8
K. pneumoniae GTH-A15
K. pneumoniae GTH-A1
K. pneumoniae GTH-A2
K. pneumoniae GTH-A20
K. pneumoniae GTH-A13
K. pneumoniae GTH-A5
K. pneumoniae GTH-A6
K. pneumoniae GTH-A18
K. pneumoniae GTH-A11
K. pneumoniae GTH-A9
K. pneumoniae GTH-A10
E. hormaechei GTH-H10
E. hormaechei GTH-E11
K. pneumoniae GTH-G3
E. cloacae GTH-I3
K. pneumoniae GTH-H7
Pantoea sp. GTH-G15
K. variicola GTH-G11
E. hormaechei GTH-F13
K. pneumoniae GTH-G4
E. coli GTH-H9
E. coli GTH-H8
K. aerogenes GTH-F8
K. aerogenes GTH-H11
V. parahaemolyticus GTH-H4

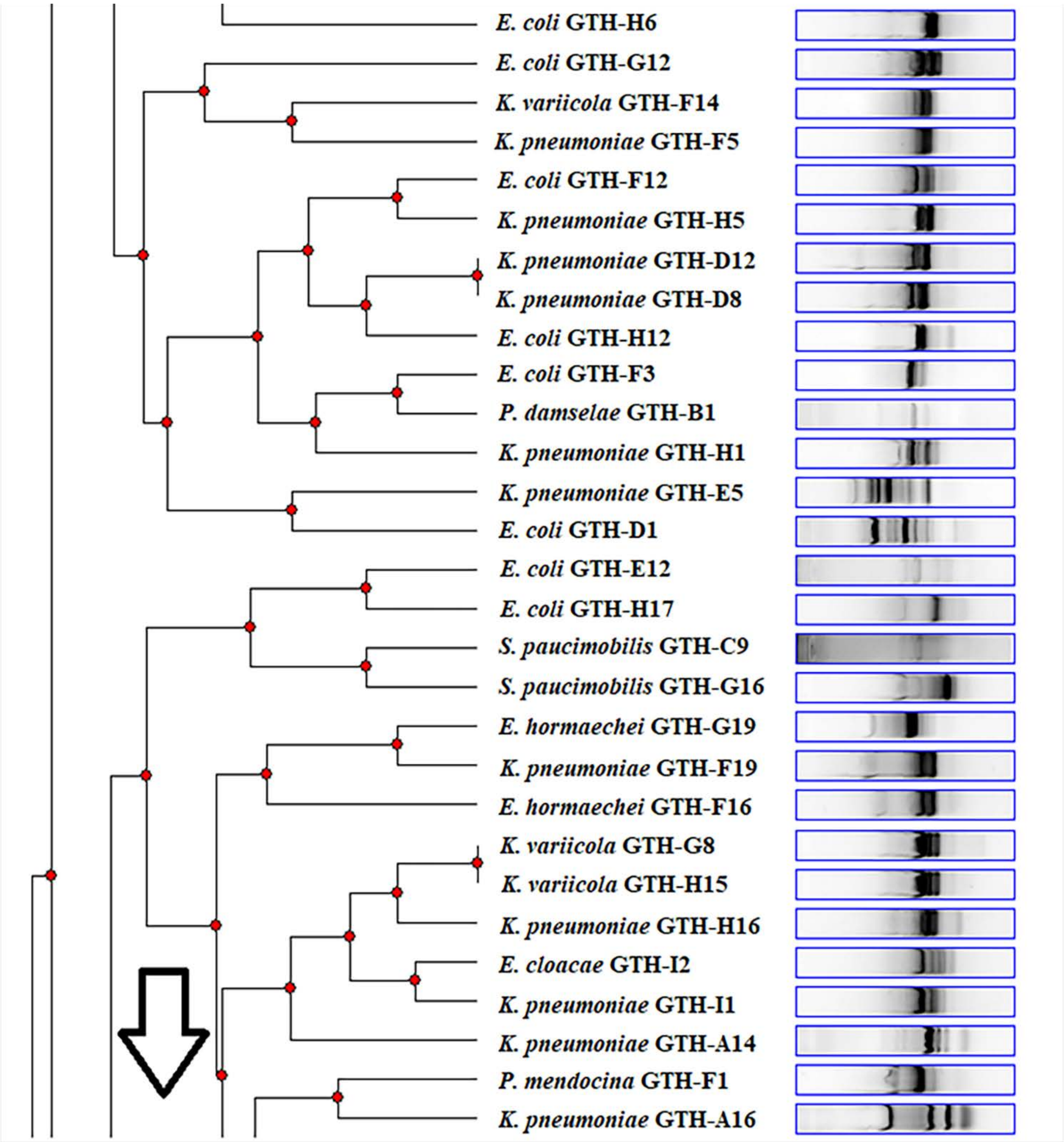

E. coli GTH-H6
E. coli GTH-G12
K. variicola GTH-F14
K. pneumoniae GTH-F5
E. coli GTH-F12
K. pneumoniae GTH-H5
K. pneumoniae GTH-D12
K. pneumoniae GTH-D8
E. coli GTH-H12
E. coli GTH-F3
P. damselae GTH-B1
K. pneumoniae GTH-H1
K. pneumoniae GTH-E5
E. coli GTH-D1
E. coli GTH-E12
E. coli GTH-H17
S. paucimobilis GTH-C9
S. paucimobilis GTH-G16
E. hormaechei GTH-G19
K. pneumoniae GTH-F19
E. hormaechei GTH-F16
K. variicola GTH-G8
K. variicola GTH-H15
K. pneumoniae GTH-H16
E. cloacae GTH-I2
K. pneumoniae GTH-I1
K. pneumoniae GTH-A14
P. mendocina GTH-F1
K. pneumoniae GTH-A16

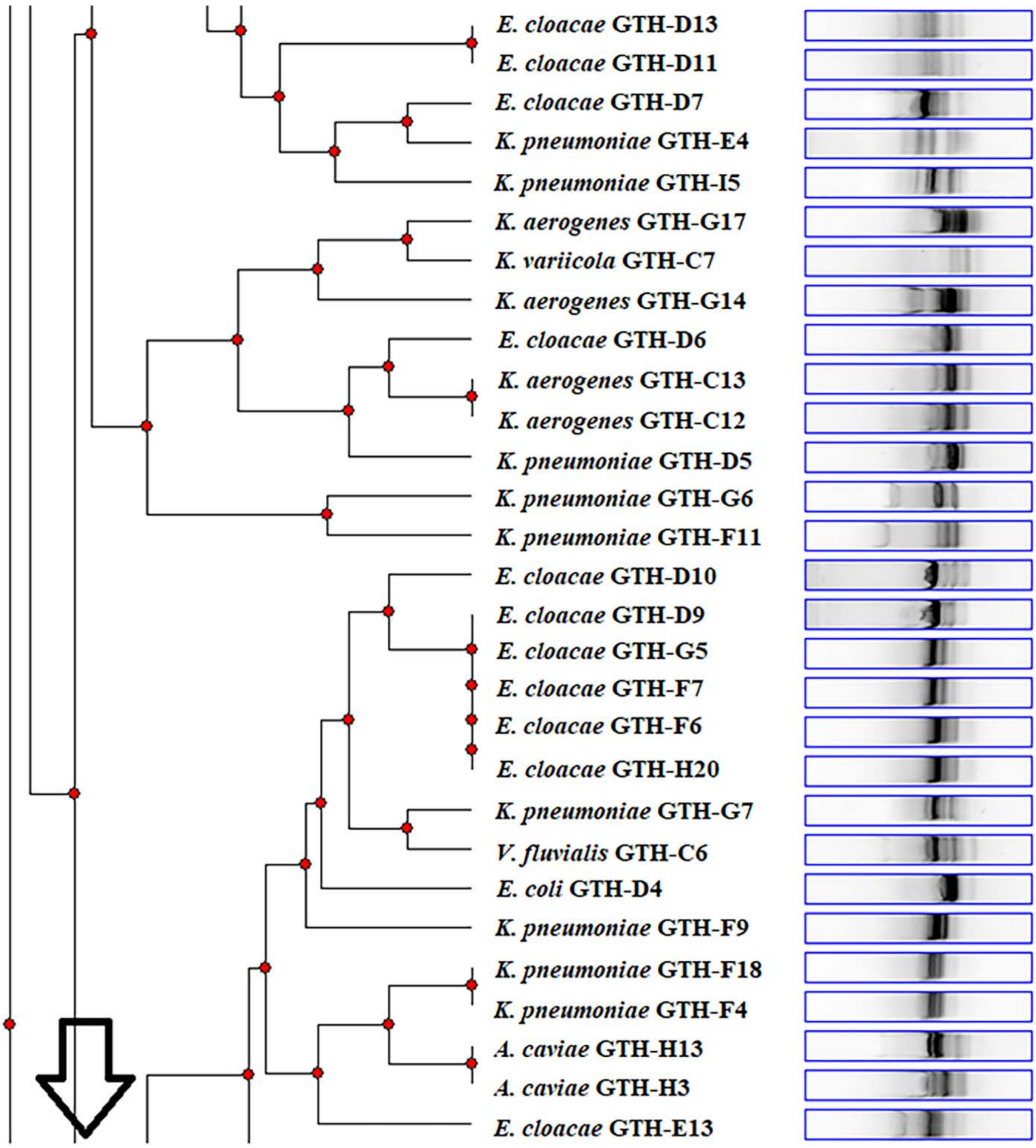

E. cloacae GTH-D13
E. cloacae GTH-D11
E. cloacae GTH-D7
K. pneumoniae GTH-E4
K. pneumoniae GTH-I5
K. aerogenes GTH-G17
K. variicola GTH-C7
K. aerogenes GTH-G14
E. cloacae GTH-D6
K. aerogenes GTH-C13
K. aerogenes GTH-C12
K. pneumoniae GTH-D5
K. pneumoniae GTH-G6
K. pneumoniae GTH-F11
E. cloacae GTH-D10
E. cloacae GTH-D9
E. cloacae GTH-G5
E. cloacae GTH-F7
E. cloacae GTH-F6
E. cloacae GTH-H20
K. pneumoniae GTH-G7
V. fluvialis GTH-C6
E. coli GTH-D4
K. pneumoniae GTH-F9
K. pneumoniae GTH-F18
K. pneumoniae GTH-F4
A. caviae GTH-H13
A. caviae GTH-H3
E. cloacae GTH-E13

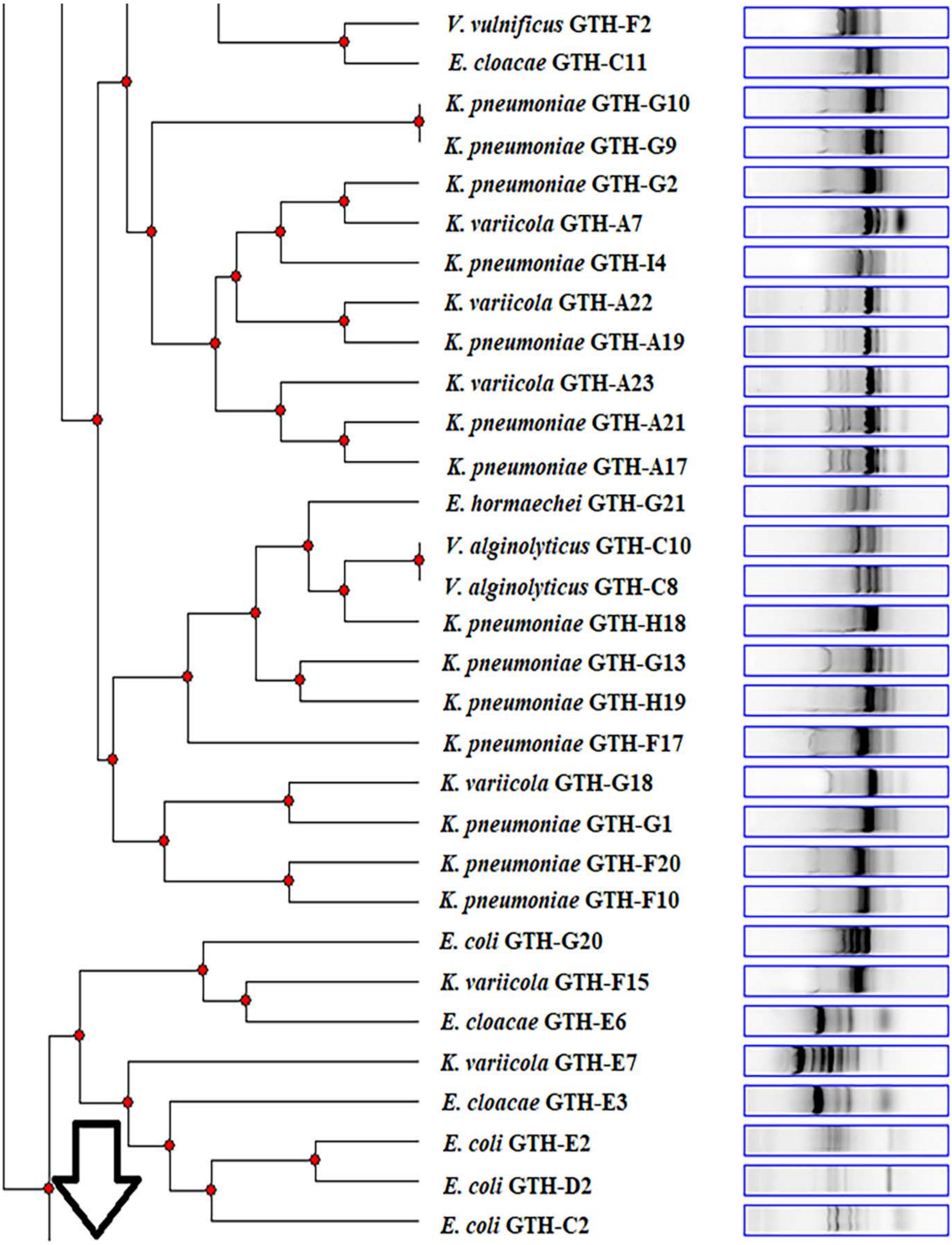
V. vulnificus GTH-F2
E. cloacae GTH-C11
K. pneumoniae GTH-G10
K. pneumoniae GTH-G9
K. pneumoniae GTH-G2
K. variicola GTH-A7
K. pneumoniae GTH-I4
K. variicola GTH-A22
K. pneumoniae GTH-A19
K. variicola GTH-A23
K. pneumoniae GTH-A21
K. pneumoniae GTH-A17
E. hormaechei GTH-G21
V. alginolyticus GTH-C10
V. alginolyticus GTH-C8
K. pneumoniae GTH-H18
K. pneumoniae GTH-G13
K. pneumoniae GTH-H19
K. pneumoniae GTH-F17
K. variicola GTH-G18
K. pneumoniae GTH-G1
K. pneumoniae GTH-F20
K. pneumoniae GTH-F10
E. coli GTH-G20
K. variicola GTH-F15
E. cloacae GTH-E6
K. variicola GTH-E7
E. cloacae GTH-E3
E. coli GTH-E2
E. coli GTH-D2
E. coli GTH-C2

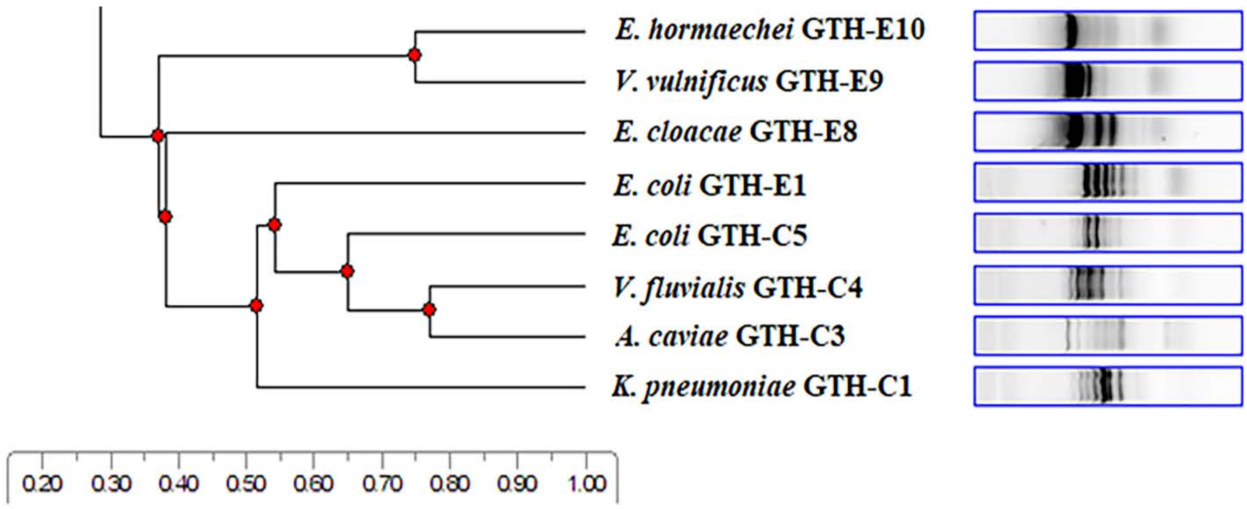

Figure S1. Dendrogram constructed from ERIC-PCR patterns of the 129 presumptive enterobacterial isolates.
