# Supplementary material for: Identification, Characterization, and Virulence Gene Expression of Marine Enterobacteria in the Upper Gulf of Thailand
Source: Microorganisms. 2022 Feb 26;10(3):511. doi: 10.3390/microorganisms10030511 (PMC8952428; doi:10.3390/microorganisms10030511)
Supplement: Supplementary file 1 [file microorganisms-10-00511-s001.zip › Table S1 R1.pdf]

**Table S1**. Nucleotide sequences of primers and sizes of the PCR products for the examined antibiotic resistance and virulence genes.

| Gene | Nucleotide sequence of primer | Size of the PCR product (bp) | Reference |
|---|---|---|---|
| *ampC* | AMPCf 5' ATT CGT ATG CTG GAT CTC GCC ACC 3' | 395 | [64] |
| | AMPCr 5' CAT GAC CCA GTT CGC CAT ATC CTG 3' | | |
| *blaCMY2* | CMY2f 5' TGA TGC AGG AGC AGG CTA TTC CA 3' | 323 | [65] |
| | CMY2r 5' CTA ACG TCA TCG GGG ATC TGC 3' | | |
| *blaCTX-M* | CTXMf 5' ATG TGC AGY ACC AGT AAR GTK ATG GC 3' | 593 | [66] |
| | CTXMr 5' TGG GTR AAR TAR GTS ACC AGA AYC AGC GG 3' | | |
| *blaKPC* | KPCf 5' ATG TCA CTG TAT CGC CGT C 3' | 863 | [65] |
| | KPCr 5' AAT CCC TCG AGC GCG AGT 3' | | |
| *blaNDM* | NDMf 5' GGT GCA TGC CCG GTG AAA TC 3' | 660 | [65] |
| | NDMr 5' ATG CTG GCC TTG GGG AAC G 3' | | |
| *blaSHV* | SHVf 5' CTT TAT CGG CCC TCA CTC AA 3' | 237 | [66] |
| | SHVr 5' AGG TGC TCA TCA TGG GAA AG 3' | | |
| *blaTEM* | TEMf 5' CGC CGC ATA CAC TAT TCT CAG AAT GA 3' | 445 | [66] |
| | TEMr 5' ACG CTC ACC GGC TCC AGA TTT AT 3' | | |
| *blaVIM* | VIMf 5' GTT TGG TCG CAT ATC GCA AC 3' | 382 | [65] |
| | VIMr 5' AAT GCG CAG CAC CAG GAT AGA A 3' | | |
| *blaZ* | BLAZf 5' TTA AAG TCT TAC CGA AAG CAG 3' | 377 | [67] |
| | BLAZr 5' TAA GAG ATT TGC CTA TGC TT 3' | | |
| *mecA* | MECAf 5' CCT AGT AAA GCT CCG GAA 3' | 314 | [68] |
| | MECAr 5' CTA GTC CAT TCG GTC CA 3' | | |
| *cnf2* | CNF2f 5' GTG AGG CTC AAC GAG ATT ATG CAC TG 3' | 839 | [69] |

| | | | |
|---|---|---|---|
| | CNF2r 5’ CCA CGC TTC TTC TTC AGT TGT TCC TC 3’ | | |
| *csgD* | CSGDf 5’ TGA AAR YTG GCC GCA TAT CAA TG 3’ | 355 | [70] |
| | CSGDr 5’ ACG CCT GAG GTT ATC GTT TGC C 3’ | | |
| *eaeA* | EAEAf 5’ TGA GCG GCT GGC ATG AGT CAT AC 3’ | 241 | [69] |
| | EAEAr 5’ TCG ATC CCC ATC GTC ACC AGA GG 3’ | | |
| *espB* | ESPBf 5’ GCC GCT CTG ATT GGT GGT GCT 3’ | 387 | [71] |
| | ESPBr 5’ TGG CGT TGA ACC GGA AAT CCT 3’ | | |
| *kfu* | KFUf 5’ GAA GTG ACG CTG TTT GTG GC 3’ | 797 | [72] |
| | KFUr 5’ TTT CGT GTG GCC AGT GAC TC 3’ | | |
| *LTI* | LTIf 5’ TGG ATT CAT CAT GCA CCA CAA GG 3’ | 360 | [69] |
| | LTIr 5’ CCA TTT CTC TTT TGC CTG CCA TC 3’ | | |
| *magA* | MAGAf 5’ GGT GCT CTT TAC ATC ATT GC 3’ | 1,280 | [72] |
| | MAGAr 5’ GCA ATG GCC ATT TGC GTT AG 3’ | | |
| *STII* | STIIf 5’ CCC CCT CTC TTT TGC ACT TCT TTC C 3’ | 423 | [69] |
| | STIIr 5’ TGC TCC AGC AGT ACC ATC TCT AAC CC 3’ | | |
| *uge* | UGEf 5’ TCT TCA CGC CTT CCT TCA CT 3’ | 534 | [72] |
| | UGEr 5’ GAT CAT CCG GTC TCC CTG TA 3’ | | |
| *vt2e* | VT2Ef 5’ CCA GAA TGT CAG ATA ACT GGC GAC 3’ | 322 | [69] |
| | VT2Er 5’ GCT GAG CAC TTT GTA ACA ATG GCT G 3’ | | |
