# Supplementary material for: Identification, Characterization, and Virulence Gene Expression of Marine Enterobacteria in the Upper Gulf of Thailand
Source: Microorganisms. 2022 Feb 26;10(3):511. doi: 10.3390/microorganisms10030511 (PMC8952428; doi:10.3390/microorganisms10030511)
Supplement: Supplementary file 1 [file microorganisms-10-00511-s001.zip › Table S2.pdf]

Table S2. Antibiotic resistance patterns of enterobacterial strains and numbers of strain(s) belonging to each pattern.

Antibiotic resistance pattern	Number of resistant strain(s) (%)	Antibiotic resistance pattern	Number of resistant strain(s) (%)	Antibiotic resistance pattern	Number of resistant strain(s) (%)
1) none	6 (5.9%)	2) TI	3 (3.0%)	3) CX	6 (5.9%)
4) CIP	1 (1.0%)	5) GEN	1 (1.0%)	6) TOB	2 (2.0%)
7) AMP TI	15 (14.9%)	8) AMP CX	9 (8.9%)	9) CX NX	1 (1.0%)
10) AMP TI CX	1 (1.0%)	11) AMP TI CIP	6 (5.9%)	12) AMP TI NX	1 (1.0%)
13) AMP CX CIP	1 (1.0%)	14) TI CIP NX	1 (1.0%)	15) AMP TI CX CIP	1 (1.0%)
16) AMP TI CX NX	1 (1.0%)	17) AMP TI CX C	1 (1.0%)	18) AMP TI CPM CIP	2 (2.0%)
19) AMP TI CPM NX	1 (1.0%)	20) AMP TI CIP NX	13 (12.9%)	21) AMP TI CIP AK	1 (1.0%)
22) AMP TI NX TOB	1 (1.0%)	23) AMP TI NX C	2 (2.0%)	24) TI CIP NX TOB	1 (1.0%)
25) AMP TI CX CTR C	2 (2.0%)	26) AMP TI CX CPM NX	1 (1.0%)	27) AMP TI CPM CIP NX	2 (2.0%)
28) AMP TI CIP LE NX	1 (1.0%)	29) AMP TI CIP NX TOB	1 (1.0%)	30) TI CX CIP NX C	1 (1.0%)
31) AMP TI CX CPM CIP C	1 (1.0%)	32) AMP TI CX CPM AK C	1 (1.0%)	33) AMP TI CTR CIP NX C	1 (1.0%)
34) AMP TI MRP CIP LE NX	1 (1.0%)	35) AMP TI CPM CIP NX TOB	1 (1.0%)	36) AMP TI CIP NX GEN TOB	1 (1.0%)
37) AMP CX MRP CIP NX AK	1 (1.0%)	38) AMP TI CIP LE NX TOB C	1 (1.0%)	39) AMP TI CIP NX AK GEN TOB	1 (1.0%)
40) AMP TI CIP NX AK TOB C	1 (1.0%)	41) TI CX CPM MRP NX TOB C	1 (1.0%)	42) AMP TI CX CIP LE NX TOB C	1 (1.0%)
43) AMP TI CPM CIP NX GEN TOB C	1 (1.0%)	44) AMP TI CX CIP NX AK GEN TOB C	1 (1.0%)	45) AMP TI CX CTR CPM CIP LE NX AK GEN TOB	1 (1.0%)

AK: amikacin (30 μg); AMP: ampicillin (10 μg); C: Chloramphenicol (30 μg); CIP: ciprofloxacin (5 μg); CPM: cefepime (30 μg); CTR: ceftriaxone (30 μg); CX: cefoxitin (30 μg); GEN: gentamicin (10 μg); LE: levofloxacin (5 μg); MRP: meropenem (10 μg); NX: norfloxacin (10 μg); TI: ticarcillin (75 μg); TOB: tobramycin (10 μg)
